# Supplementary figures and images for: Population sequencing reveals clonal diversity and ancestral inbreeding in the grapevine cultivar Chardonnay
Source: PLoS Genet. 2018 Nov 20;14(11):e1007807. doi: 10.1371/journal.pgen.1007807 (PMC6279053; doi:10.1371/journal.pgen.1007807)

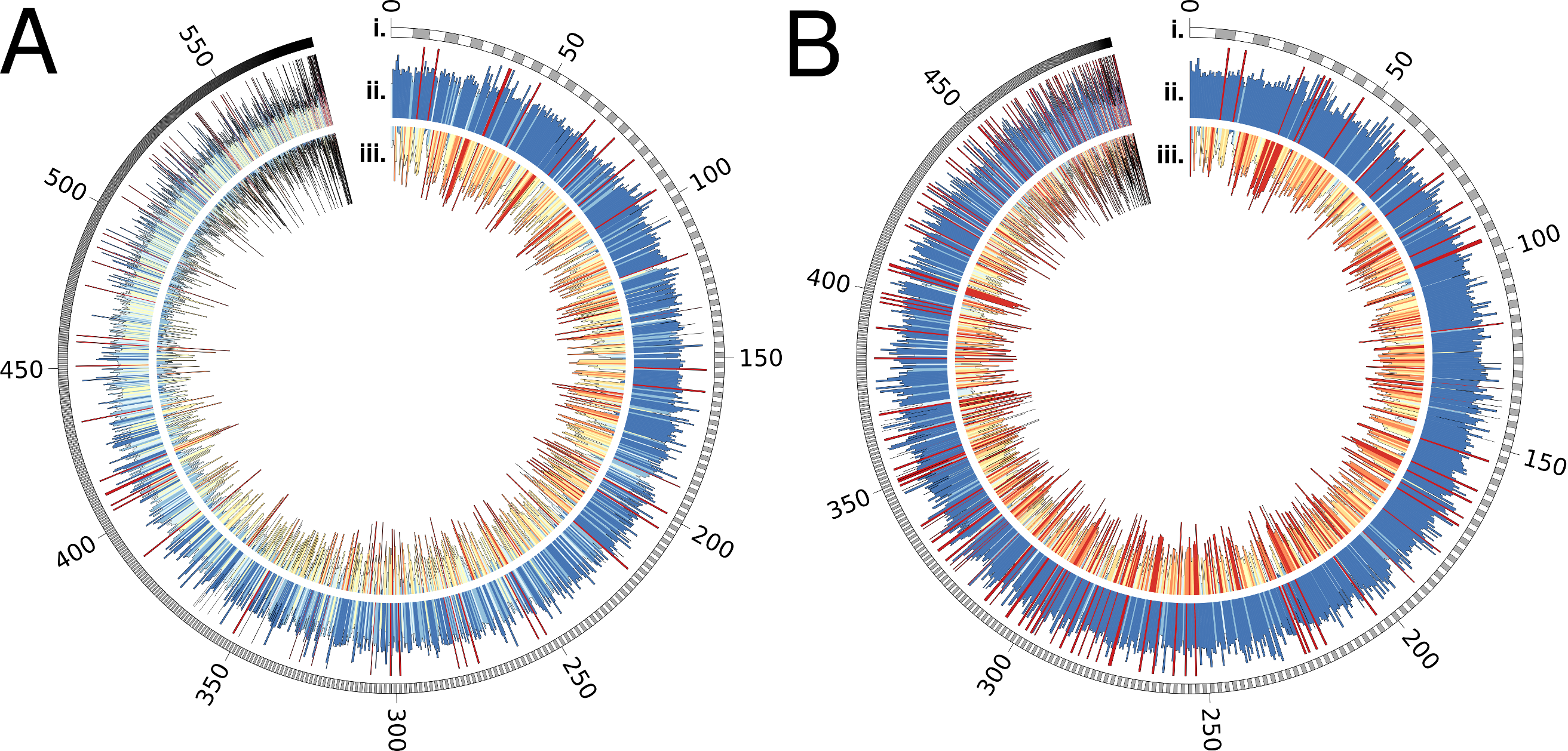

Supplement: S1 Fig — (A) Circular representations of FALCON Unzip Chardonnay assembly and (B) the same assembly after curation. Tracks are: length-ordered contigs (i), read depth of mapped PacBio RS II subreads, coloured by read-depth (blue, high; yellow, median; red, low) (ii) and heterozygous SNP density, coloured by SNP density (blue, low; yellow, median; red, high) (iii). (TIFF) [file pgen.1007807.s003.tiff]

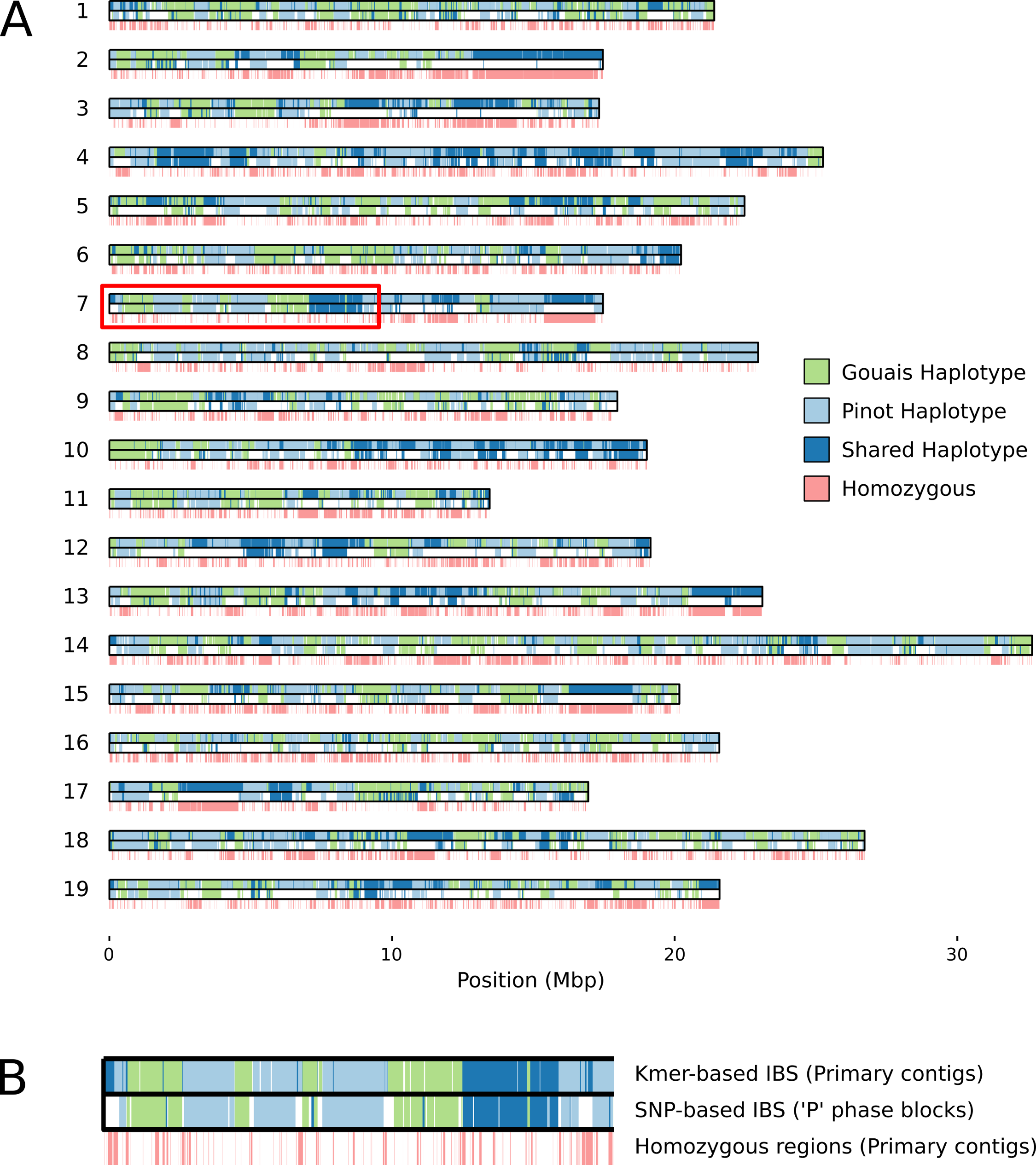

Supplement: S2 Fig — (A) an ideogram of the Chardonnay reference assembly with the positions of primary contigs (but not haplotigs) for both the kmer- and SNP-based IBS methods, juxtaposed with homozygous annotations (fewer than 10 SNPs per 5 kb window). Gaps in phase-blocks are indicated in white. (B) An enlargement of a region of Vitis vinifera Chromosome 7 (red box in A). (TIFF) [file pgen.1007807.s004.tiff]

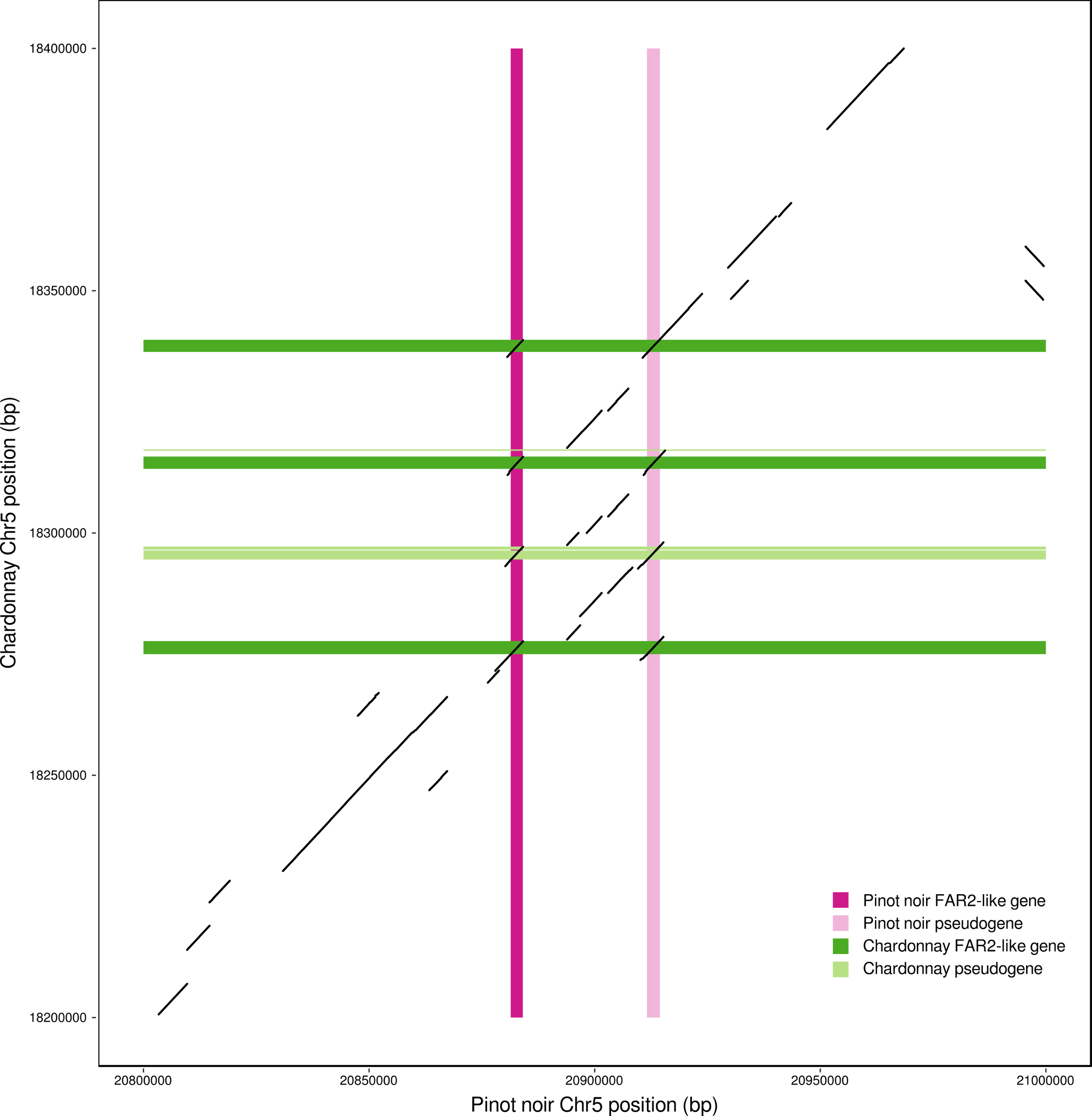

Supplement: S3 Fig — Alignments are indicated as black lines (dotplot), the ORFs for FAR2-like genes and pseudogenes are indicated for both Pinot noir and Chardonnay. (TIFF) [file pgen.1007807.s005.tiff]

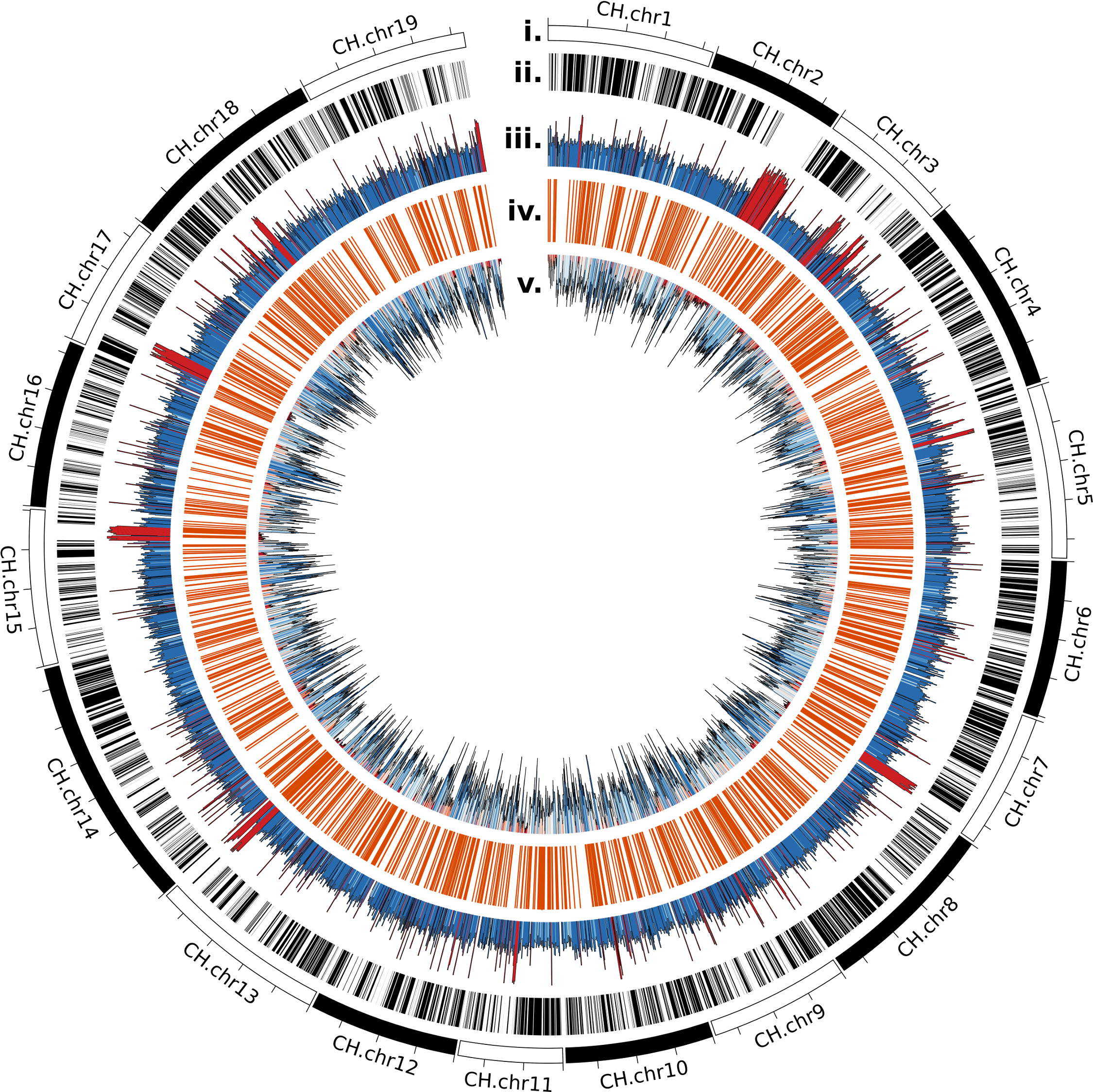

Supplement: S4 Fig — Circos plot derived from Fig 1A with the inclusion of the clonal marker locations. The tracks shown are chromosome-ordered primary contigs (i), haplotig alignments (ii), read-depth of RS II subreads mapped to diploid assembly (read-depth colour scale: yellow, low; blue, high; red, double) (iii), locations of clonal markers (iv), and heterozygous variant density (SNP density colour scale: red, low; blue, high) (v). (TIFF) [file pgen.1007807.s006.tiff]
