# Supplementary material for: Population sequencing reveals clonal diversity and ancestral inbreeding in the grapevine cultivar Chardonnay
Source: PLoS Genet. 2018 Nov 20;14(11):e1007807. doi: 10.1371/journal.pgen.1007807 (PMC6279053; doi:10.1371/journal.pgen.1007807)
Supplement: S1 Table — (PDF) [file pgen.1007807.s007.pdf]

S1 Table: **BUSCO analysis of the Chardonnay FALCON Unzip assembly before and after curation.**

		Primary Contigs		Haplotigs		Primary contigs + Haplotigs	
		#	%	#	%	#	%
	Total BUSCO groups searched	1440	100	1440	100	1440	100
FALCON Unzip	Complete BUSCOs	1277	88.68	1002	69.58	1348	93.61
	Complete and single-copy BUSCOs	1177	81.74	959	66.60	691	47.99
	Complete and duplicated BUSCOs	100	6.94	43	2.99	657	45.63
	Fragmented BUSCOs	31	2.15	56	3.89	21	1.46
	Missing BUSCOs	132	9.17	382	26.53	71	4.93
Curated	Complete BUSCOs	1290	89.58	1093	75.90	1347	93.54
	Complete and single-copy BUSCOs	1230	85.42	1060	73.61	690	47.92
	Complete and duplicated BUSCOs	60	4.17	33	2.29	657	45.63
	Fragmented BUSCOs	43	2.99	58	4.03	23	1.60
	Missing BUSCOs	107	7.43	289	20.07	70	4.86
