# Supplementary material for: Population sequencing reveals clonal diversity and ancestral inbreeding in the grapevine cultivar Chardonnay
Source: PLoS Genet. 2018 Nov 20;14(11):e1007807. doi: 10.1371/journal.pgen.1007807 (PMC6279053; doi:10.1371/journal.pgen.1007807)
Supplement: S2 Table — (PDF) [file pgen.1007807.s008.pdf]

## S2 Table: Information relating to Chardonnay clones used in this study

| | Alternate name | Origin | Yield (kg/vine) | Ripening time | Comments |
|---|---|---|---|---|---|
| **CR red [34]** | | Clare, South Aust. | | | Red-coloured berries |
| **I10V1** | FPS06[81], Heat treated Wente clone | UCD (Olmo68 - FPS05) | 8.7 [82] | Early | |
| **Mendoza** | UCD1 | | 8.4 [82], low | Late | Prone to millerandage |
| **G9V7** | FPS05[1], Olmo69 (Wente or Martini selections) | Stanley Lane Vineyards, Carneros [83] | 8.5 [82], 12.9 [84], 5.2 [6] | | |
| **95** | FPS73,38 | Côte-dOr, Dijon | 6.4 [82], medium [85] | late ripening | Sour rot in northern climate |
| **76** | FPS69, FPS76 | Côte-dOr, Dijon | 5.6 [10], medium [85] | late | |
| **352** | FPS41 | Côte-dOr, l'Espiguette | 6.4 [10] | late | |
| **277** | FPS 42, 49 and 51 | Dijon | | late | |
| **96** | FPS70, FPS96 | Côte-dOr, Dijon | 6.4 [10], high [85] | late | |
| **1066** | | Côte-dOr, ENTAV | Low [85] | | Prone to millerandage |
| **124** | FPS84,98 | Côte-dOr, Champagne | 5.9 [10] | late | |
| **Waite Star [35]** | | | | | Seedless, shiny hairless leaves |
| **118** | FPS104 | Côte-dOr, Champagne | 5.9 [10] | Early | |
| **809** | | Saône-et-Loire, ENTAV | Low – medium [85] | Early | Muscat character |
| **548** | FPS548 | Saône-et-Loire, ENTAV | Low-medium [85] | late | |

## References


81. Nicholas P. Grapevine clones used in Australia. SARDI, 2006 Jan 30.
82. Cirami R, Ewart AJW. Clonal selection, evaluation and multiplication in Australia. In: Wolpert J, Walker MA, Roberts D, editors. Proceedings of the International Symposium on Clonal Selection; Sep 22; Portland, Oregon1995. p. 52-9.
83. Sweet NL. Chardonnay History and Selections at FPS. FPS Grape Program Newsletter. 2007.
84. Wolpert J, Kasimatis A, Weber E. Field performance of 6 Chardonnay clones in the Napa valley. American Journal of Enology and Viticulture. 1994;45(4):393-400.
85. Institut Français de la Vigne et du Vin (IFV) (Le Grau du Roi F, INRA Centre de Montpellier (FRA), SupAgro (Montpellier F, VINIFLHOR. Catalogue des variété et clones de vigne cultivés en france. 2ème édition: Ministère de l'agriculture de la pêche et de l'alimentation; 2007.
